# Evaluation of the Therapeutic Effect of Traditional Chinese Medicine on Osteoarthritis: A Systematic Review and Meta-Analysis

**DOI:** 10.1155/2020/5712187

**Published:** 2020-12-14

**Authors:** Lin Wang, Xiao-Fei Zhang, Xue Zhang, Dong-Yan Guo, Yu-Wei Duan, Zhi-Chao Wang, Li-Shan Pei, Han Ru, Jiang-Xue Cheng, Ya-Jun Shi, Jun-Bo Zou

**Affiliations:** ^1^Shaanxi Province Key Laboratory of New Drugs and Chinese Medicine Foundation Research, Pharmacy College, Shaanxi University of Chinese Medicine, Xianyang 712046, China; ^2^Pharmaceutical Factory of Shaanxi University of Chinese Medicine, Xianyang, China; ^3^School of Pharmacy, Health Science Center, Xi'an Jiaotong University, Xi'an, China

## Abstract

**Background:**

Osteoarthritis (OA) is a common degenerative disease of bone and joint characterized by the damage of articular cartilage and hypertonia, which often occurs in the middle-aged and elderly. Traditional Chinese medicine (TCM) therapy, including acupuncture (ACU), oral administration, and external use of traditional Chinese medicines (TCMs), can significantly improve the therapeutic effect on OA and reduce the occurrence of side effects. We provide a latest meta-analysis on the treatment of OA with TCM.

**Materials and Methods:**

In the electronic database, appropriate articles without language restrictions on keywords were selected until August 1, 2019. All trajectories are screened according to certain criteria. The quality of qualified research was also assessed. We have made a detailed record of the results of the measurement. Meta-analysis was carried out with Revman 5.3 software.

**Results:**

Forty-four articles involving 4014 patients (2012 cases in the experimental group and 2002 cases in the control group) with OA were selected. This article focuses on the study of the treatment of OA by using the general mode of TCM. The quality evaluation included in the study was evaluated independently according to the Cochrane intervention system evaluation manual. In this meta-analysis, 68.18% of the literature correctly described the conditions for the generation of random assignment sequences, only 6.82% of the literature correctly mentioned the hidden details of allocation, and all studies mentioned randomly assigned participants. Compared with Western medicine, the total effective rate (TER) of OA treatment in TCM was significantly increased and the recurrence rate (RR) was significantly decreased (*P* < 0.00001). In addition, the experimental group was also superior to the control group in terms of the indicators of joint activity function, inflammatory factor content, and various indicators affecting bone metabolism. It can be showed by the network analysis diagram that Aconiti Radix, Achyranthis Bidentatae Radix, and other TCMs can inhibit inflammatory stimulation and relieve the pain symptoms of patients with OA. ACU at Yinlingquan, Xiyan, and other acupoints can effectively improve the clinical symptoms of patients with OA.

**Conclusion:**

TCM therapy in treatment of patients with OA could effectively restore joint function, enhance the TER, and reduce RR. However, the results of this study should be handled with care due to the limitations existing. Some rigorous randomized controlled trials (RCTs) are needed to confirm these findings.

## 1. Introduction

Osteoarthritis (OA) is a chronic degenerative joint disease, which occurs frequently in the elderly, and OA is one of the diseases that lead to disability in the world [1]. The effect of this disease is significant in the knee, hip, and hands [2]. At this stage, the concept of OA has been further developed. OA is considered to be an entire joint disease, including changes in the articular cartilage, subcartilage bone, ligaments, capsule, and synovium, resulting in functional joint injury [3], resulting in joint pain and physical disability [4], thus impairing the quality of life of patients with OA.

OA is considered to be the main cause of chronic pain in clinics. The pain symptoms associated with OA lead to the decrease of physical fitness and behavior ability [5]. In the world today, the trials of pain and disability caused by OA are interdisciplinary, involving surgical treatment, drugs, and physiotherapy [6]. However, the cost of physiotherapy is important for patients, and the evidence that physiotherapy is effective in treating OA patients is not clear [7]. Although hip and knee replacement has become a routine treatment for end-stage arthritis, the risk of surgery is high, including the mortality rate caused by surgery and the failure rate of reoperation due to failed implants. Also, the cost of operation also has significant cost effectiveness [8]. The main goal of drug therapy is not only to control joint pain but also to avoid the toxic effects of treatment [9]. Nonsteroidal anti-inflammatory drugs and anesthetic painkillers are commonly used in drug therapy. However, taking these drugs may also cause gastrointestinal damage, heart and kidney load, and other side effects [10].

At present, there are no nonspecific, safe, and effective drugs and methods in clinical treatment [9], so there is an urgent need to seek a breakthrough from traditional Chinese medicine (TCM). From ancient times to the present, the use of traditional Chinese medicines (TCMs) and all kinds of TCM therapy has played an inestimable role in the treatment of OA of which experience and therapeutic effects have been tested and refined in Asian countries in the past few thousand years. OA belongs to the category of Gu Bi in TCM. In ancient times, medical saints studied OA and recorded its pathogenesis and treatment in many TCM medical works, such as “Inner Classic of the Yellow Emperor” and “Treatise on Febrile and Miscellaneous Diseases.” The most important thing is to record the excellent results of TCM in the treatment of OA [11]. The treatment of OA with TCM includes internal and external use of TCMs, acupuncture (ACU), massage manipulation and Tai Chi exercise, and other therapies. Various studies have shown that the use of TCMs has analgesic and anti-inflammatory effects on the treatment of OA. ACU is one of the most common complementary and alternative medical therapies in medicine and is also a popular therapy for relieving pain and treating dysfunction related to musculoskeletal conditions. The therapeutic effect of ACU will remain unchanged with the passage of time [12]. The use of a massage technique can effectively reduce the pain of each joint and achieve the purpose of supplementary treatment. Tai Chi exercise can improve the physical and mental health of patients and effectively relieve psychological depression symptoms caused by OA pain. This article mainly introduces the therapeutic effects of oral administration of TCMs (OATCM), external use of TCMs (EUTCM), and ACU on OA.

**Figure 1:**
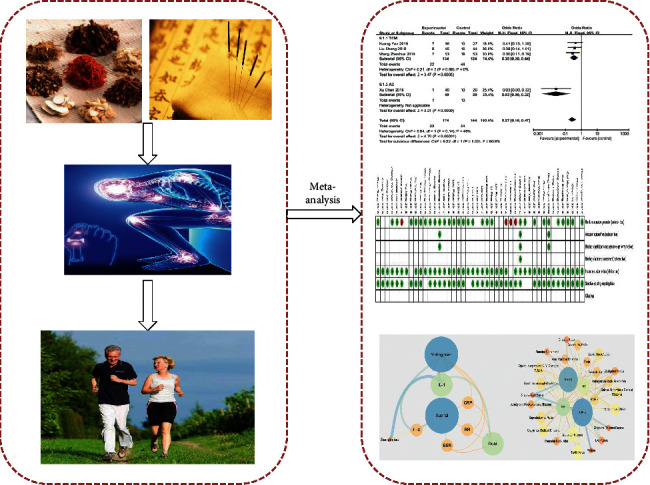
Work flow of the present study.

Here, we provide a novel and comprehensive meta analysis with detailed information for the efficacy of TCM including TCMs and ACU on patients with OA (Figure 1).

## 2. Methods and Program

As Dr. zou et al., 2019 [13], have done a lot of solid data retrieval work using various databases, their data mining and processing methods are worth learning and using for reference. Therefore, we followed the methods of Dr. Zou et al., 2019.

### 2.1. Literature Retrieval Strategy

Keywords “Osteoarthritis (OA)” [Title/Abstract] AND “Traditional Chinese medicine (TCM)” OR “Acupuncture (ACU)” [Title/Abstract] AND “Clinical” [Title/Abstract] were used as search items in electronic databases including Pubmed, Wanfang, CNKI, CBM, and VIP. Articles published before August 1, 2019, were checked without language restrictions in order to obtain a comprehensive search. All relevant articles were downloaded into Endnote software (version X9, Thomson Reuters, Inc., New York, NY, United States) for exploring further. Duplicate records were removed. A full-text review was performed while the title/abstract were thought to be thematic. The job mentioned above was executed by three investigators independently. Conflicts were resolved by the consensus and discussion.

### 2.2. Inclusion and Exclusion Criteria

According to the suggestions of medical experts, we designed the inclusion criteria as follows: (1) Patients in RCTs were diagnosed with OA by diagnostic criteria of knee osteoarthritis (DCKO), guidelines for the diagnosis and treatment of osteoarthritis (GDTO) version 2007, guiding principles for clinical research of new drugs of traditional Chinese medicine (GPCRNDTCM), clinical guideline of new drugs for traditional Chinese medicine (CGNDTCM), diagnosis and treatment scheme of traditional Chinese medicine for 95 diseases in 22 specialties (DTSTCM95D22S), criteria for diagnosis and therapeutic effect of diseases and syndromes of traditional Chinese medicine (CDTEDSTCM), or diagnostic criteria of therapeutic effect of traditional Chinese medicine on knee osteoarthritis (DCTETCMKO). (2) All trials mentioned were described as RCTs. (3) Patients in the most of the experimental groups received TCM therapy, whereas patients in the most of the control groups received Western medicine therapy. (4) Outcome measurements of each study must have included a minimum of one of the following indices: total effective rate (TER), Western Ontario and McMaster University osteoarthritis index (WOMAC), visual analogue scale (VAS), interleukin (IL)-1, IL-6, tumor necrosis factor-*α* (TNF-*α*), syndrome score of traditional Chinese medicine (SSTCM), erythrocyte sedimentation rate (ESR), *C*-reactive protein (CRP), matrix metalloproteinases-3 (MMP-3), insulin-like growth factor-1 (IGF-1), bone morphogenetic protein-7 (BMP-7), receptor activator of nuclear factor-*κ* B ligand (RANKL), bone gla protein (BGP), osteoprotegerin (OPG), fibroblast growth factor-2 (FGF-2), angiopoietin I (Ang I), vascular endothelial growth factor (VEGF), recurrence rate (RR), swelling score (SS), superoxide dismutase (SOD), transforming growth factor-*β* (TGF-*β*), Lysholm, and Lequesne.

The exclusion criterion was designed as follows: (1) Articles such as reviews, animal trials, case report, and comments were thought to be unrelated to the topic. (2) Trials were not RCTs, or diagnostic criteria in the statement were ambiguous. (3) The intervention of OA patients was not based on TCM treatment.

### 2.3. Data Extraction and Quality Assessment

Information on qualified studies, including authors, methods, sample size, interventions, and result measurements is obtained. The quality of the included study was independently evaluated by three researchers according to the Cochrane Handbook for Systematic Reviews of Interventions, and different opinions were resolved by consensus. Quality assessment includes random sequence generation (selection bias), allocation concealment (selection bias), blindness of participants and personnel (performance bias), blindness of result evaluation (detection bias), incomplete result data (attrition bias), selective reporting (reporting bias), and other deviations. There are three levels of judgment each semester. The “low risk” of the deviation indicates that the description of the method or procedure is appropriate, the “high risk” indicates that the description of the method or procedure is insufficient or incorrect, and the “unclear risk” indicates that the description of the method and/or procedure is insufficient.

### 2.4. Data Analysis

Data analysis was performed using Review Manager 5.3 (Cochrane Collaboration). Outcome measures such as TER and RR were regarded as dichotomous variables and presented as the odds ratio (OR) with 95% confidence intervals (95% CI), contents of inflammatory cytokines (IL-1, IL-6, TNF-*α*, MMP-3, and TGF-*β*), indexes of scoring (SSTCM, VAS, WOMAC, Lysholm, SS, and Lequesne), and levels of bone metabolism (IGF-1, OPG, BGP, FGF-2, RANKL, and BMP-7), and factors of ESR and CRP were continuous variables that presented as the mean difference (MD) with 95% CI. *P* statistics and *I*^2^ tests were applied to assess heterogeneity among studies. A fixed-effect model was used to analyze data with low heterogeneity (*P* > 0.1 and *I*^2^ ≤ 50%), and data with high heterogeneity (*P* < 0.1 or *I*^2^ > 50%) were estimated using the random-effects model. Potential publication bias was revealed by funnel plots.

## 3. Results

### 3.1. Characteristics of the Eligible Studies

**Figure 2:**
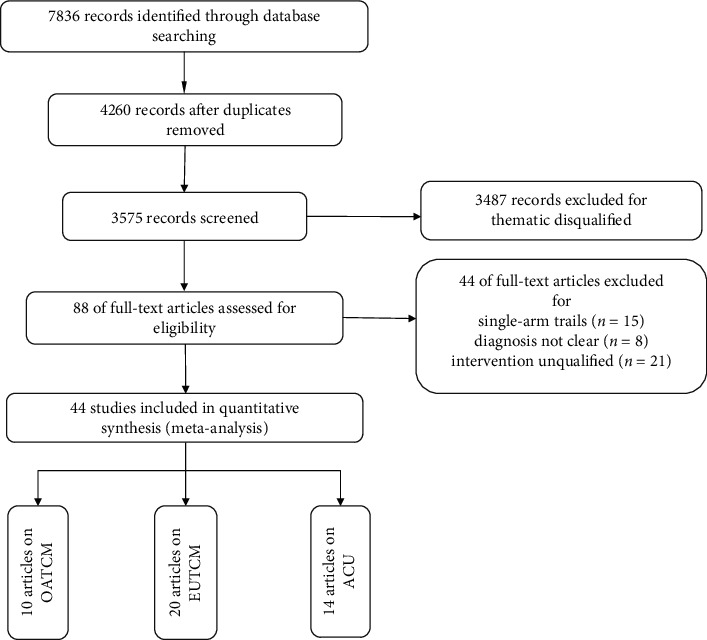
Process of the study extracted for meta-analysis.

A total of 7836 studies were identified through database retrieval, of which 4260 were deleted due to duplication. Of the remaining 3575 studies, 3487 were excluded due to substandard topics. After that, there were still 88 articles to be further examined in full. Forty-four studies were excluded from this procedure because of unclear diagnosis in 8 articles, inappropriate interventions in 21 studies, and single-arm design in 15 studies. Forty-four studies [14–57] were included in quantitative synthesis finally (Figure 2).

Meta-analysis was performed on 4014 OA patients (2012 in the experimental group and 2002 in the control group). The age of the patients ranged from 40 to 80 years, and there was no obvious difference in terms of age and sex between the two groups (Table 1). Trials were conducted between 2000 and 2019; all were RCTs with a comparison between TCM therapy only and Western medicine therapy or with a comparison between a combination of TCM therapy and Western medicine therapy. The usual regimens of TCM therapy were OATCM, EUTCM, and the ACU. In the EUTCM, it can be divided into the methods of traditional Chinese medicine iontophoresis (TCMI), hot compress of TCMs, and fumigation of TCMs. The usual regimens of Western medicine therapy were sodium hyaluronate (SH), arthroscopic debridement (AD), and Western medicine capsules. Thirty studies reported that the duration of treatment lasted for 2 to 4 weeks. Three trials reported a follow-up ranging from 3 to 6 months (Table 2). The TCMs and ACU involved in each article were integrated. The prescriptions handed down from ancient times to today, including the OATCM and EUTCM group, were sorted out (Table S1). The acupoint involved in the ACU group articles were also integrated (Table S2). At the same time, we provided the corresponding international code for acupoint (Table S3).

### 3.2. Quality of Included Trials Assessment

**Figure 3:**
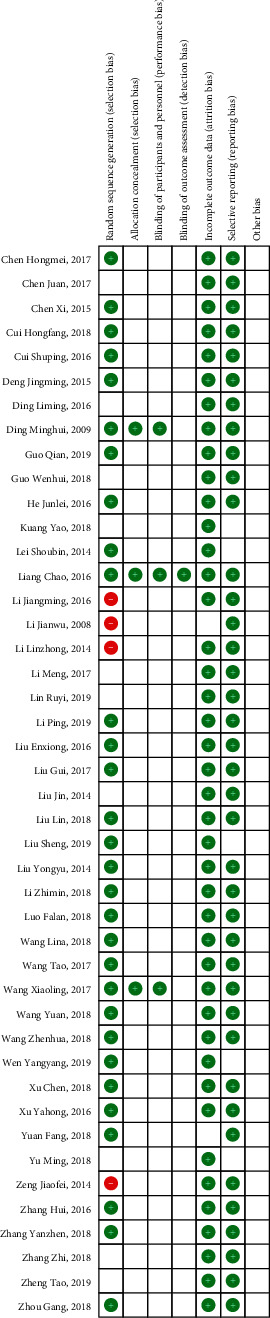
Risk of bias assessment in eligible studies. The quality assessment was conducted by Review Manager 5.3 according to Cochrane Handbook for Systematic Reviews of Interventions Version 5.1.0. Red circle, high risk of bias; green circle, low risk of bias; blank, unclear risk of bias.

According to the Cochrane risk of deviation estimation, all trials in the literature mentioned the random allocation of participants, and 30 trials [14, 16–18, 20, 21, 23–26, 28, 29, 32–34, 38–46, 51–53, 55–57] described the conditions for the generation of random allocation sequences. Detailed information on allocation concealment and blinding of participants of majority studies was mentioned in 3 trials [45, 56, 57]. Blinding of outcome assessment of all studies was reported in 1 trial [56]. Forty-two studies [14–36, 38–50, 52 –57] were considered to have a lower risk of wear bias due to the availability of complete data. Thirty-nine trials [14–18, 21–24, 27–29, 31–57] reported detailed indicators, thus indicating a lower risk of reporting bias (Figure 3).

### 3.3. Outcome Measures with Subgroup Analysis

#### 3.3.1. TER of TCM Therapy vs. Western Medicine Therapy

**Figure 4:**
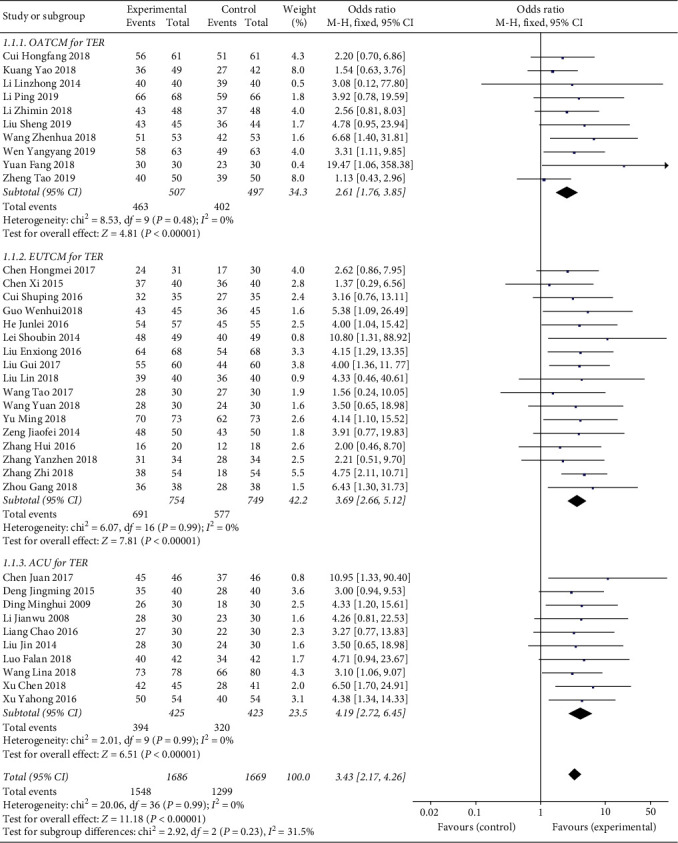
Forest plot of TER in patients treated with TCM therapy and Western medicine therapy. I2 and P are the criteria for the heterogeneity test. ◆: pooled odds ratio, —■—: odds ratio, and 95%CI.

The standard of clinical efficacy reported in the included trials was divided into four grades including clinical recovery, marked effect, effectiveness, and invalidation. TER referred to the percentage of patients who were evaluated to recovery, marked effect, and effectiveness. Thirty-seven studies reported TER among which there were ten trials [14, 16, 19, 20, 26, 29, 39, 47, 48, 51] in the OATCM group which meta-analyzed that OATCM treatment significantly improved TER in the treatment of OA (OR = 2.61, 95%CI: 1.76, 3.85; *P* < 0.00001) using a fixed-effect model (*P*=0.99, *I*^2^ = 0% for heterogeneity test). Seventeen studies [15, 17, 22–25, 30, 33, 34, 36, 38, 40, 41, 43, 46, 52, 55] demonstrated that EUTCM treatment significantly improved TER (OR = 3.69, 95%CI: 2.66, 5.12; *P* < 0.00001) using a fixed-effect model (*P*=0.99, *I*^2^ = 0% for heterogeneity test). Ten trials [21, 32, 35, 37, 42, 44, 50, 53, 56, 57] mentioned that ACU treatment improved TER significantly (OR = 4.19, 95%CI: 2.72, 6.45; *P* < 0.00001) with a fixed-effect model (*P*=0.99, *I*^2^ = 0% for heterogeneity test) of meta-analysis. According to the overall analysis of TER, TCM therapy significantly improved the clinical efficiency compared with the control group (Figure 4).

#### 3.3.2. Indicators of Self-Activity Score of TCM Therapy vs. Western Medicine Therapy

VAS, WOMAC, SSTCM, Lysholm, SS, and Lequesne were the important indices mentioned in included studies reflecting self-activity and therapeutic effects.

**Figure 5:**
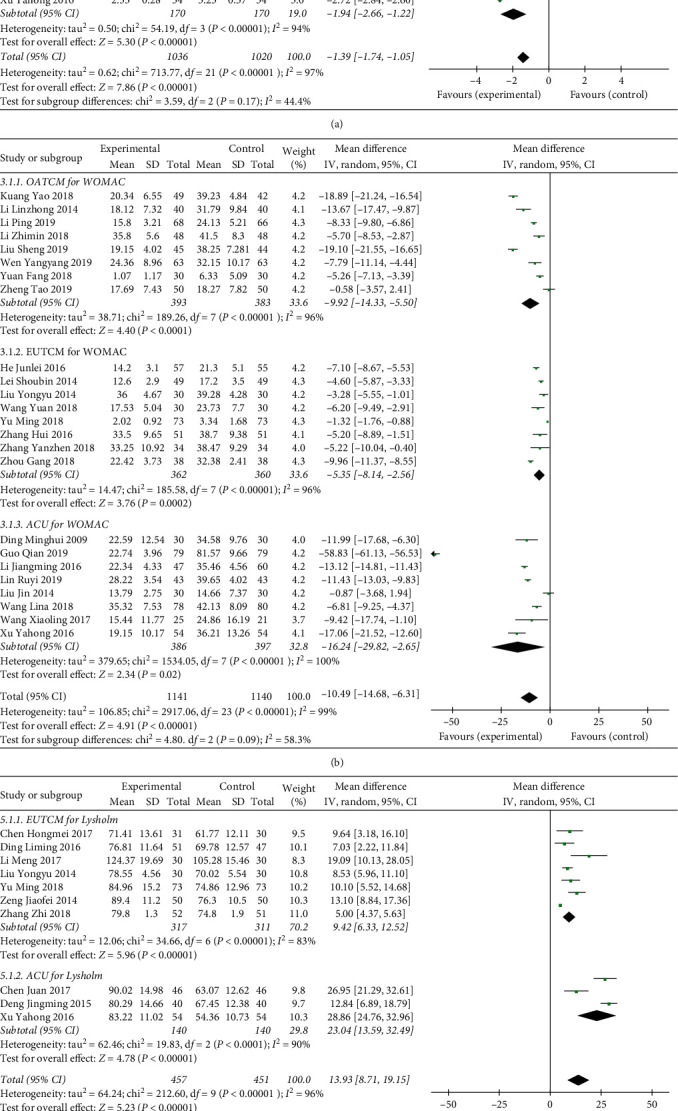
Forest plot of self-activity score in patients treated with TCM therapy and Western medicine therapy. (a) The plot of VAS, (b) the plot of WOMAC, (c) the plot of Lysholm, and (d) the plot of SSTCM. (I)^2^ and (P) are the criteria for the heterogeneity test, ◆: pooled mean difference, —■—: mean difference, and 95%CI.

Seven trials [14, 16, 19, 20, 26, 47, 51] mentioned the determination of VAS in the OATCM group (MD = −1.45, 95%CI: −2.05, −0.85; *P* < 0.00001) with heterogeneity (*P* < 0.00001, *I*^2^ = 97%). Eleven trials [15, 17, 24, 30, 31, 34, 36, 38, 43, 49, 55] recorded VAS in the EUTCM group (MD = −1.12, 95%CI: −1.58, −0.67; *P* < 0.00001) with heterogeneity (*P* < 0.00001, *I*^2^ = 95%), and four trials [21, 37, 50, 53] provided VAS in the ACU group (MD = −1.94, 95%CI: −2.66, −1.22; *P* < 0.00001) with heterogeneity (*P* < 0.00001, *I*^2^ = 94%), so a random-effect model was applied to finish the meta-analysis mentioned above. The result of meta-analysis demonstrated that the TCM therapy significantly decreased the level of VAS (MD = -1.39, 95%CI: −1.74, −1.05; *P* < 0.00001; Figure 5(a)).

In terms of WOMAC index, it was reported in the OATCM group, EUTCM group, and ACU group. Eight trials [14, 19, 20, 26, 29, 47, 48, 51] mentioned the WOMAC in the OATCM group (MD = −9.92, 95%CI: −14.33, −5.50; *P* < 0.0001) with heterogeneity (*P* < 0.00001, *I*^2^ = 96%). Eight trials [17, 18, 25, 30, 34, 41, 52, 55] recorded WOMAC in the EUTCM group (MD = −5.35, 95%CI:−8.14, −2.56; *P* *=* 0.0002) with heterogeneity (*P* < 0.00001, *I*^2^ = 96%), and eight trials [21, 27, 28, 32, 35, 45, 54, 57] reported in the ACU group (MD = −16.24, 95%CI: −29.82, −2.65; *P*=0.02) with heterogeneity (*P* < 0.00001, *I*^2^ = 100%), so a random-effect model was applied to finish the meta-analysis mentioned above which showed that the TCM therapy significantly decreased the level of WOMAC compared with the control group (MD = −10.49, 95%CI: −14.68, −6.31; *P* < 0.00001; Figure 5(b)).

In terms of the Lysholm score, seven trials [15, 18, 30, 31, 36, 38, 49] recorded Lysholm in the EUTCM group (MD = 9.42, 95%CI: 6.33, 12.52; *P* < 0.00001) with heterogeneity (*P* < 0.00001, *I*^2^ = 83%) and three trials [21, 50, 53] provided Lysholm in the ACU group (MD = 23.04, 95%CI: 13.59, 32.49; *P* < 0.00001) with heterogeneity (*P* < 0.00001, *I*^2^ = 90%), so a random-effect model was applied to finish the meta-analysis mentioned above which demonstrated that the TCM therapy significantly increased the level of Lysholm (MD = 13.93, 95%CI: 8.71, 19.15; *P* < 0.00001; Figure 5(c)).

In terms of the SSTCM, three trials [16, 39, 51] recorded SSTCM in the OATCM group (MD = −4.01, 95%CI: −5.15, −2.86; *P* < 0.00001) with heterogeneity (*P*=0.01, *I*^2^ = 76%) and three trials [33, 52, 55] provided SSTCM in the EUTCM group (MD = -1.95, 95%CI: −3.74, −0.17; *P*=0.03) with heterogeneity (*P*=0.005, *I*^2^ = 81%), so a random-effect model was applied to finish the meta-analysis mentioned above which showed that the TCM therapy significantly decreased the level of SSTCM (MD = −3.06, 95%CI: −4.16, −1.95; *P* < 0.00001; Figure 5(d)).

#### 3.3.3. Inflammatory Cytokines of TCM Therapy vs. Western Medicine Therapy

**Figure 6:**
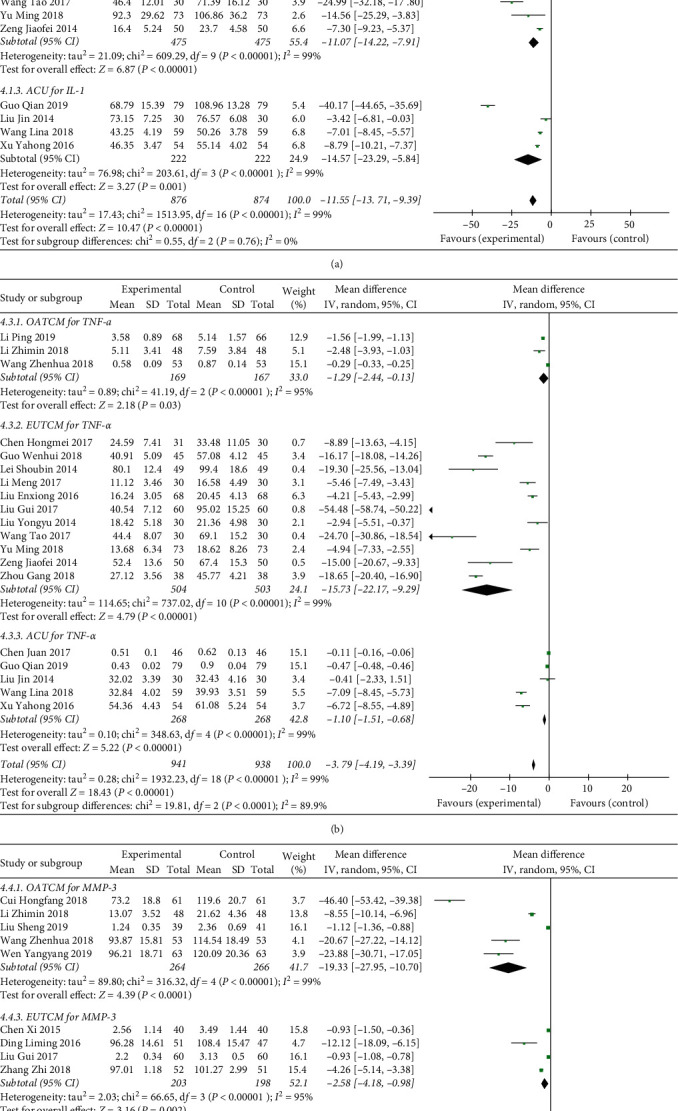
Forest plot of inflammatory cytokines in patients treated with TCM therapy and Western medicine therapy. (a) The plot of IL-1, (b) the plot of TNF-*α*, and (c) the plot of MMP-3. (I)^2^ and (P) are the criteria for the heterogeneity test. ◆: pooled mean difference, —■—: mean difference, and 95%CI. In the detection of inflammatory factors, due to differences in treatment methods, treatment cycles, detection environments, detection instruments, and detection personnel, the detection values vary greatly, but the directions are the same. Therefore, this indicator can be evaluated by the evaluation manager 5.3.

Inflammatory cytokines play an important role in the occurrence and development of OA. Inflammatory indices reported in eligible studies included IL-1, IL-6, TNF-*α*, MMP-3, and TGF-*β*. For the IL-1 index, three studies [14, 20, 29] reported in the OATCM group with meta-analysis that OATCM significantly decreased the level of IL-1 (MD = −11.54, 95%CI: −25.51, 2.42) with a random-effect model because of heterogeneity existence (*P* < 0.00001, *I*^2^ = 99%). Ten articles [18, 22, 23, 25, 30, 31, 36, 40, 43, 46] proved that EUTCM therapy significantly decreased the level of IL-1 (MD = −11.07, 95%CI: −14.22, −7.91; *P* < 0.00001) using a random-effect model because of heterogeneity existence (*P* < 0.00001, *I*^2^ = 99%). Four trials [21, 28, 32, 35] mentioned that ACU therapy can significantly decrease the IL-1 (MD = −14.57, 95%CI: −23.29, −5.84; *P*=0.001) with a random-effect model (*P* < 0.00001, *I*^2^ = 99% for the heterogeneity test) of meta-analysis (Figure 6(a)).

Three studies [14, 16, 29] in the OATCM group, eleven trials [18, 22, 23, 25, 30, 31, 36, 38, 40, 41, 43] in the EUTCM group, and five trials [21, 28, 32, 35, 50] in the ACU group reported that TCM therapy reduced the level of TNF-*α* significantly of which MD with 95%CI was ((MD = −1.29, 95%CI: −2.44, −0.13; *P*=0.03), (MD = −15.73, 95%CI: −22.17, −9.29; *P* < 0.00001), and (MD = −1.10, 95%CI: −1.51, −0.68; *P* < 0.00001)) meta-analyzed by the random-effect model (*P* < 0.00001, *I*^2^ = 99% for the heterogeneity test, respectively) (Figure 6(b)).

Five studies [14, 16, 20, 26, 39] of OATCM, four trials [15, 40, 46, 49] of EUTCM, and one article [27] of the ACU group mentioned the MMP-3. The MD with 95% CI for MMP-3 was ((MD = −19.33, 95%CI: −27.95, −10.70), (MD = −2.58, 95%CI: −4.18, −0.98), and (MD = −25.44, 95%CI: −30.28, −20.60)) certified a significant decrease in the experimental group compared with control group (*P* < 0.0001; Figure 6(c)).

#### 3.3.4. Influence on Other Indices of TCM Therapy vs. Western Medicine Therapy

In this meta-analysis, we summarized 5 and less articles related to the same index and summarized the results in the supplemental files. OATCM therapy can significantly reduce SS (*P* < 0.0001) and Lequesne (*P* < 0.00001) levels in self-activity indicators of patients with OA. Also, EUTCM therapy can also reduce Lequesne (*P* < 0.0001), but the therapeutic effect of SS (*P*=0.27) has no statistical significance (Table S4). Associated inflammatory factors include TGF-*β* and IL-6. OATCM treatment could significantly increase the level of TGF-*β* (*P* < 0.00001), but had no statistical significance in reducing IL-6 (*P*=0.12). Both EUTCM therapy and ACU therapy can significantly increase TGF-*β* and decrease IL-6 levels (*P* < 0.001) (Table S5). Among the indicators related to bone balance, ACU therapy can significantly increase the level of BGP and FGF-2 and reduce RANKL (*P* < 0.001), but there is no statistical significance for the improvement of IGF-1 (*P*=0.24) and OPG (*P*=0.62). OATCM therapy can significantly reduce BMP-7 (*P* < 0.00001) (Table S6). ACU therapy can significantly reduce the levels of Ang I and VEGF (*P* < 0.00001) in the collected studies (Table S7). OATCM and EUTCM therapy can effectively reduce the RR and increase SOD (*P* < 0.00001) (Table S8). A forest plot illustrating the results of the analysis of ESR and CRP indicators is shown in Figure S1. Both OATCM and ACU therapy can effectively reduce ESR and CRP (*P* < 0.00001). In addition, EUTCM therapy can significantly reduce ESR (*P*=0.09), but there is no statistical significance for CRP (*P*=0.15).

### 3.4. Network Relationship Diagram between Acupoints and Corresponding Indexes

**Figure 7:**
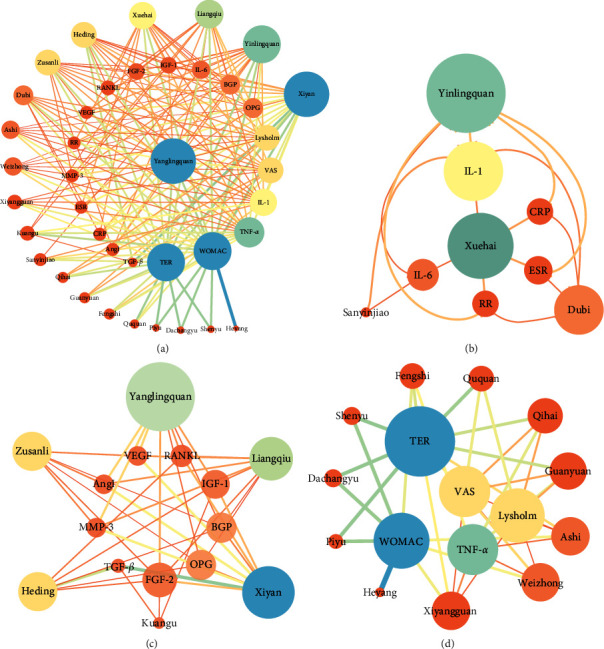
Corresponding relationship between the acupoint and index network. The larger nodes are represented by the main correspondence.

The 21 acupoints and 19 related indexes were imported into Cytoscape 3.7.1 software, and the network analysis of the interaction between acupoints and corresponding indexes in ACU treatment of OA was drawn, Figure 7(a), through the module analysis of the main graph using the eagle algorithm through ClusterViz plug-in Cytoscape, and three main relational subgraphs were obtained. Figure 7(a) shows the overall relationship between acupoints and indicators. Figure 7(b) shows the interaction between Yinlingquan (ST9) and Xuehai (SP10) acupoints and inflammatory factors, which have major therapeutic effects. Figure 7(c) mainly shows the effects of acupoint Yanglingquan (GB34), Liangqiu (ST34), Xiyan (EX-LE4), Zusanli (ST36), and Heding (EX-LE2) on FGF-2, OPG, and BGP of the bone metabolism indexes. Figure 7(d) shows the effect of acupoints such as Xiyangguan (GB33), Weizhong (BL40), and Ashi on the level of TER, WOMAC, and VAS. After ACU treatment, the indexes have been well improved, and the symptoms of OA have been significantly improved (Figure 7).

### 3.5. Network Relationship Diagram between TCMs and Corresponding Indexes

**Figure 8:**
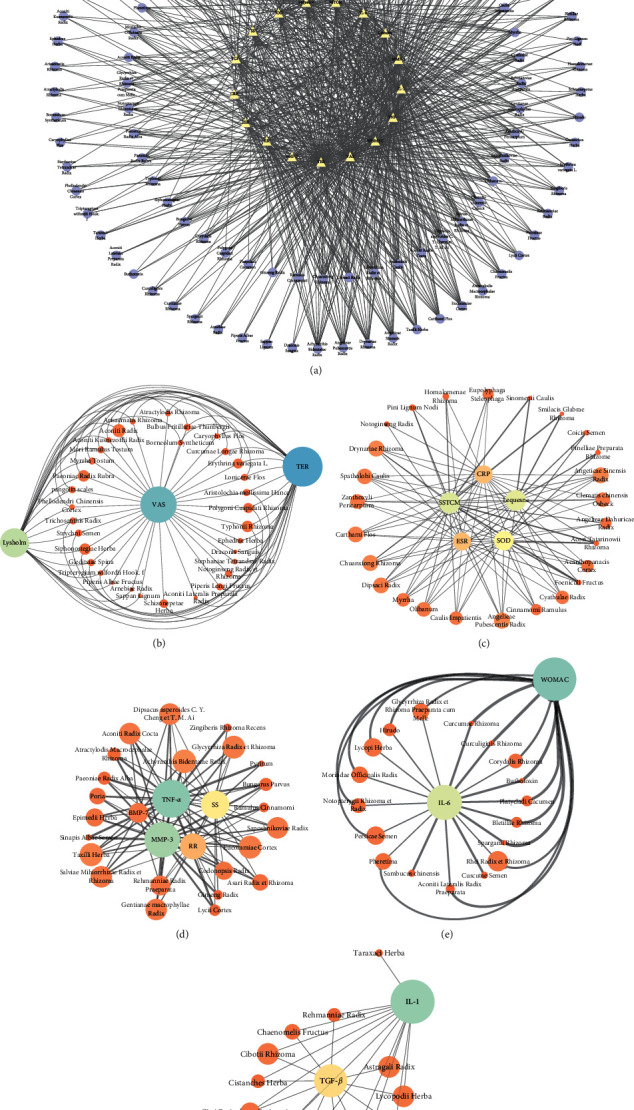
Corresponding relationship between TCMs and the index network. The larger nodes are represented by the main correspondence.

The 113 TCMs and 17 related indexes were imported into Cytoscape 3.7.1 software, and the network analysis diagram of the interaction between TCMs and indicators interaction in the treatment of OA was drawn, as shown in Figure 8(a). Then, through ClusterViz plug-in Cytoscape, the module analysis of the main graph is carried out by using EAGLE algorithm to get five main relational subgraphs. Figure 8(a) shows the relationship between TCMs and indicators. Figure 8(b) shows that TCMs such as Dipsaci Radix, Aconiti Radix, Typhonii Rhizoma, and Siphonostegiae Herba can ameliorate the score of the pain scale and improve the clinical efficacy. Figure 8(c) shows that Achyranthis Bidentatae Radix, Chuanxiong Rhizoma, Carthami Flos, Angeticae Sinensis Radix, and other drugs can effectively reduce ESR and CRP inflammation indicators ESR and CRP, reduce pain, and improve joint function. Figure 8(d) shows that TCMs such as Drynariae Rhizoma, and Taxilli Herba have a significant relationship with inflammation, reducing SS by reducing TNF-*α* and MMP-3. Figure 8(e) shows that Rhei Radix et Rhizoma, Lycopi Herba, and Persicae Semen can effectively reduce the content of IL-6 and then reduce the WOMAC. Figure 8(f) shows that Lycopodii Herba, Astragali Radix, and Liquidambaris Fructus significantly reduce the content of IL-1 in OA, indicating that these TCMs have a significant effect on the treatment of patients with OA (Figure 8).

### 3.6. Publication Bias

**Figure 9:**
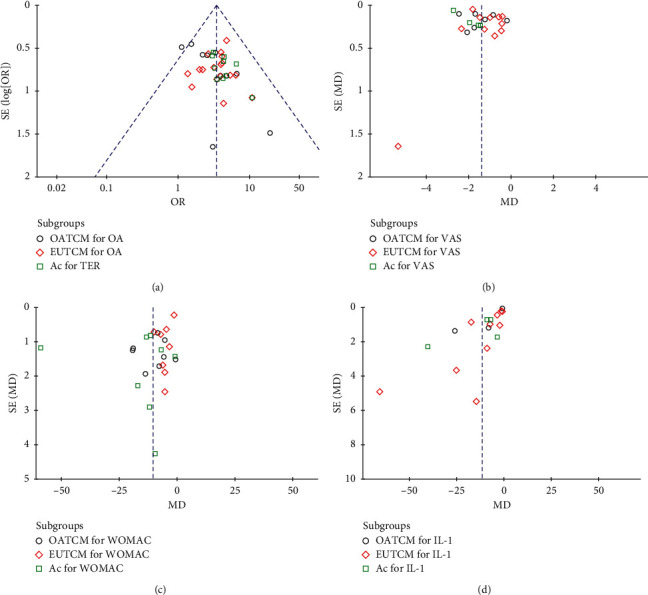
Funnel plot for the publication bias. (a) The plot of TER, (b) the plot of VAS, (c) the plot of WOMAC, and (d) the plot of IL-1.

Publication bias was expressed by a funnel plot. In this study, funnel plots of TCM therapy vs. Western medicine therapy on TER, VAS, WOMAC, and IL-1 were applied. The plot is basically symmetrical except that there is some bias due to different usage methods of TCMs in the EUTCM group, which indicates that there is no obvious publication bias (Figures 9(a)–9(d)).

## 4. Discussion

OA is a degenerative disease that causes pain, stiffness, and decline in body function [6]. With the combined effects of ageing and increasing obesity in the global population, along with increasing numbers of joint injuries, the occurrence of diseases such as OA is becoming more prevalent, with worldwide estimates suggesting that 250 million people are currently affected [58]. At this stage, there are great risks for the various diagnosis and treatment effects of Western medicine, and there is a lack of high-quality data on the benefits and hazards of adverse drug reactions [59]. Current treatments for OA are inadequate [60]. In the theory of TCM, OA belongs to the category of osteoarthralgia, which has been recorded in various literature. The Chinese medicine is through the classification of the causes of OA, symptomatic administration, and the use of appropriate methods of treatment [34]. In the treatment of OA, the use of TCM has few side effects and remarkable curative effects. In contemporary development, the treatment of TCM will be more and more popular among the public.

The results of this meta-analysis show that the treatment of TCM has a significant TER for OA and could reduce the RR. Moreover, there were significant differences in WOMAC (*P* < 0.00001), VAS (*P* < 0.00001), IL-1 (*P* < 0.00001), TNF-*α* (*P* < 0.00001), BGP (*P*=0.007), and other indicator factors in patients with OA treated by TCM. It could regulate the expression of inflammatory factors, improve the synthesis and invasion of reactive oxygen species on chondrocytes collagen, reduce cartilage damage, block bone destruction, balance bone absorption and bone metabolism, reduce pain in patients, and promote the body's self-activity function [61–64].

ACU is an effective physiotherapy for the treatment of OA with few side effects [65]. The compatibility of multiple acupoints focuses on dispelling wind and activating collaterals, eliminating arthralgia, and relieving pain [66]. Combined with the usual treatment of OA and the acupoints summed up by the network pharmacological map, it can be concluded that Yinlingquan (ST9), Xiyan (EX-LE4), Weizhong (BL40), Xuehai (SP10), Yanglingquan (GB34), and Zusanli (ST36) have a great effect on the treatment of OA. According to the analysis of the relationship between TCMs and the index network, Aconiti Radix, Achyranthis Bidentatae Radix, Typhonii Rhizoma, Persicae Semen, and Chuanxiong Rhizoma are very effective in the treatment of OA. The use of these TCMs and acupoints can not only significantly reduce inflammatory factors but also reduce pain and improve the quality of life of patients.

After a comprehensive analysis of the included literature, it was found that there were some limitations: 1. Although the 44 included studies were all RCTs, most of them did not describe the allocation scheme and allocation concealment in detail, which may lead to selection bias and implementation bias. 2. In this included experiment, all the studies were in Chinese, which may make the data collection incomplete and, thus, may have a biased effect on the analysis results. 3. The literature included in this paper lacks uniformity in reference standards for total efficiency and does not strictly distinguish their differences, which may affect the reliability of the results. Therefore, it is suggested that the criteria of clinical efficacy should be unified in future studies. 4. There are deviations in the quality and objectivity of the literature in the selection of research. In addition, the clinical risk assessment and report of the included literature are not clear, which may have a certain impact on the demonstration strength of TCM efficacy. 5. The indicators included in this meta-analysis have some heterogeneity, which may have an impact on the accuracy and objectivity of the results. Therefore, in the future systematic review and meta-analysis, more large samples and high-quality RCTs should be included. It is also hoped that the later RCTs can clarify the specific methods of randomized trials, and more rigorous clinical trials can be conducted to improve the reliability of the clinical effects of TCM.

Compared with other articles, this meta-analysis adopted a more systematic literature retrieval method and included a large sample size. It also contained more comprehensive outcome measures, making this meta-analysis more complete and reliable. With the help of the network relationship construction model in network pharmacology, network analysis was conducted on all TCMs, acupoints, and corresponding indicators involved in this meta-analysis, so as to find the most core relationship between TCM diagnosis and treatment of OA.

## 5. Conclusions

The use of various treatments of TCM can significantly improve the level of TER and reduce the occurrence of RR in the later stage of treatment. These effects are mediated by a combination of several mechanisms. This method of treatment with TCM can reduce pain, improve their ability to move, and improve their living standards by reducing the level of VAS, WOMAC, SSTCM, SS, and Lequesne and increasing the level of Lysholm. TCM therapy can reduce inflammation and exert anti-inflammatory effects by reducing the levels of IL-1, IL-6, TNF-*α*, MMP-3, ESR, and CRP and increasing TGF-*β*. TCM therapy can increase BGP, FGF-2, IGF-1, and OPG and reduce BMP-7 and RANKL levels to improve bone metabolism in order to achieve the balance of bone metabolism. The TCM therapy can also reduce the levels of VEGF and Ang I, reduce cartilage injury, and restore vascular endothelial function. The effect of TCM therapy can also increase the level of SOD and reduce the existence of reactive oxygen species. The net analysis of ACU and index showed that ACU at local acupoints such as Yinlingxue, Xuehai, and Yanglingquan can reduce inflammatory indexes, reduce cartilage damage, balance bone metabolism, reduce WOMAC, and improve TER. The network analysis chart of TCMs and index showed that TCMs can effectively reduce WOMAC through the inhibition of IL-1 and MMP-3 and improve the effect of TER and so on. However, our findings must be handled with care because of the small size and low quality of the clinical trial samples cited. Other rigorous and large-scale RCTs are needed to confirm these results.

## Figures and Tables

**Table 1:** Characteristics of eligible studies.

	Author, year	Cases *T*/C	Diagnostic standard	Age (years) range, mean		Sex male/female	
OATCM	Wang Zhenhua, 2018	53/53	GDTO and GPCRNDTCM	T: 41–72, 50.17	C: 43–74, 51.49	T:29/24	C: 31/22
	Kuang Yao, 2018	49/42	GDTO and DTSTCM95D22S	T: 56.13	C: 55.65	T: 25/24	C: 20/22
	Wen Yangyang, 2019	63/63	GDTO and GPCRNDTCM	T: 61.98	C: 62.61	T: 32/31	C: 36/27
	Cui Hongfang, 2018	61/61	GDTO and GPCRNDTCM	T: 45–76, 64.7	C: 46–75, 62.5	T:31/31	C: 32/29
	Zheng Tao, 2019	50/50	GDTO and CDTEDSTCM	T: 52–67, 63.26	C: 51–67	T:27/23	C: 24/26
	Yuan Fang, 2018	30/30	GDTO (2007) and CDTEDSTCM	T: 42–72, 54.47	C: 41–72, 60.63	T: 4/26	C: 6/24
	Li Zhimin, 2018	48/48	DCKO and CDTEDSTCM	T: 47–78, 57.42	C: 45–76, 58.24	T: 29/19	C: 31/17
	Liu Sheng, 2019	50/50	GDTO (2010) and TCMDTS95D22S	T: 54–70, 58.34	C:53–71, 57.43	T: 25/25	C: 26/24
	Li Ping, 2019	74/74	CETCMDTKO	T: 47–73, 60.69	C: 45–75, 59.48	T: 24/50	C: 25/49
	Li Linzhong, 2014	40/40	NR	T: 42–75, 57.43	C:41–74, 56.31	32/48	—

EUTCM	Yu Ming, 2018	73/73	DCKO (2007)	43–80, 54.86	—	T: 40/33	C: 46/27
	Li Meng, 2017	30/30	DCKO	T: 41–61, 52.41	C: 42–60, 51.27	T: 13/17	C: 12/18
	Lei Shoubin, 2014	49/49	GDTO	T: 43–70, 57.5	C: 44–71, 58.7	T: 17/32	C: 18/31
	Zeng Jiaofei, 2014	50/50	DCKO and K–L	T:45–63, 56.1	C:45–65, 55.6	T: 21/29	C: 23/27
	He Junlei, 2016	57/55	DCKO	T: 45–73, 58.61	C: 43–75, 59.06	T: 16/41	C:19/36
	Chen Xi, 2015	40/40	DCKO and GDTO	40–75, 61.2	—	35/45	—
	Liu Enxiong, 2016	68/68	GDTO and GPCRNDTCM	T: 53–79, 61.29	C: 51–78, 61.01	T: 45/23	C: 48/20
	Zhou Gang, 2018	38/38	GDTO (2007) and CDTEDSTCM	T: 46–70, 55.62	C: 45–68, 54.33	T: 16/22	C: 20/18
	Lui Gui, 2017	60/60	GDTO and GPCRNDTCM	T: 59–72, 64.14	C: 58–74, 64.29	T: 19/41	C: 21/39
	Ding Liming, 2016	51/47	NR	T: 63.00	C: 67.08	T: 16/35	C: 14/33
	Zhang Yanzhen, 2018	34/34	CDTEDSTCM and DCKO (1995)	T: 42–73, 52.34	C: 42–73, 54.24	T: 12/22	C: 10/24
	Liu Yongyu, 2014	30/30	DGDTO	T: 51–69	C: 51–69	NR	NR
	Wang Yuan, 2018	30/30	DCKO (1995)	T: 40–74, 58.67	C:44–73, 58.03	T: 8/22	C: 2/28
	Zhang Zhi, 2018	52/51	GDTO (2007) and K–L	T: 55–68, 63.9	C: 53–70, 64.8	T: 24/28	C: 24/27
	Chen Hongmei, 2017	31/30	DCKO	T: 58.18	C:55.90	T: 13/18	C: 11/19
	Zhang Hui, 2016	51/51	PO and TCMDTS95D22S	T: 41–79, 57.2	C: 40–78, 56.1	T: 17/34	C:19/32
	Liu Lin, 2018	40/40	DCCASTCM	T: 40–70	C: 40–70	NR	NR
	Cui Shuping, 2016	35/35	GDTO and GPCRNDTCM	T: 42–78	C: 46–80	T: 14/21	C: 12/20
	Wang Tao, 2017	30/30	GPCRNDTCM and GDTO &TCMDTS95D22S	T: 42–57, 51.1	C: 43–56, 50.2	T: 8/22	C: 9/21
	Guo Wenhui, 2018	45/45	DCKA (2007)	T: 73.4	C: 72.7	T: 25/20	C: 21/24

ACU	Chen Juan, 2017	46/46	GDTO (2007) and GPCRNDTCM	T: 45–76, 58.51	C: 47–80, 59.23	T: 25/21	C: 27/19
	Deng Jingming, 2015	40/40	GDTO (2007)	T: 60.0	C: 62.0	T: 17/23	C: 15/25
	Ding Minghui, 2009	30/30	DCKO (1986)	T: 59.37	C: 58.93	T: 8/22	C:8/22
	Guo Qian, 2019	79/79	GDTO and DCTETCMKO	T: 45–78, 56.06	C:45–78, 56.06	T: 38/41	C: 38/41
	Li Jianwu, 2008	30/30	DCKO (1995)	T: 53.0	C: 55.6	T: 11/19	C:9/21
	Li Jiangming, 2016	47/60	NR	T: 65.12	C: 64.26	T: 5/42	C: 7/53
	Liang Chao, 2016	30/30	GDTO (2007)	T: 61.4	C: 60.3	T: 22/8	C: 21/9
	Lin Ruyi, 2019	43/43	GDTO (2007)	T: 40–69, 60.05	C:40–69, 59.52	T: 22/21	C: 24/19
	Liu Jin, 2014	30/30	GDTO (2007)	T: 60.8	C: 62.5	T: 12/18	C: 13/17
	Luo Falan, 2018	42/42	GDTO (2007) and GPCRNDTCM	T: 49–67, 59.27	C: 51–68, 58.45	T: 18/24	C: 19/23
	Wang Lina, 2018	59/59	NR	T: 40–75, 52.13	C: 41–77, 53.47	T: 24/35	C: 25/34
	Wang Xiaoling, 2017	25/21	DCKO (1995)	T: 50–74, 61	C: 44–75, 58	T: 8/17	C:2/19
	Xu Chen, 2018	45/41	DCKO (1995)	T: 61.0	C: 61.0	T: 24/21	C: 25/16
	Xu Yahong, 2016	54/54	GDTO and GPCRNDTCM	T: 45–73, 54.3	C: 45–75, 55.2	T: 25/29	C:24/30

*C*, control group; CDTEDSTCM, criteria for diagnosis and therapeutic effect of diseases and syndromes of traditional Chinese medicine; DCKO, diagnostic criteria of knee osteoarthritis; DTSTCM95D22S, diagnosis and treatment scheme of traditional Chinese medicine for 95 diseases in 22 specialties; DCTETCMKO, diagnostic criteria of therapeutic effect of traditional Chinese medicine on knee osteoarthritis; GDTO, guidelines for the diagnosis and treatment of osteoarthritis; GPCRNDTCM, guiding principles for clinical research of new drugs of traditional Chinese medicine; K–L, Kellgren–Lawwrence grading standard; NR, no report; PO, practical orthopaedics; *T*, trial group.

**Table 2:** Intervention characteristics of included studies.

	Study ID (name, year)	Intervention		Duration/follow-up	Outcome measures
		Trial group	Control group		
OATCM	Wang Zhenhua, 2018	TCM-1,150 ml, bid + *C*	OI, 13 ml, qw + DSESRC, 1tablet, qd	2 weeks/1 year	TER, VAS, TNF- *α*, IL-6, MMP-3, BMP-7, SSTCM, and RR
	Kuang Yao, 2018	TCM-2,150 ml, bid + Ac	GSC, 0.5 g, tid	4 weeks/6 months	TER, VAS, WOMAC, SS, and RR
	Wen Yangyang, 2019	TCM-3, bid + TCMT	CC_1_, 200 mg,qd + TCMT	4 weeks/NR	TER, VAS, WOMAC, IL-1, TGF-*β*, MMP-3, and SOD
	Cui Hongfang, 2018	TCM-4,150 mg, bid + *C*	DSSRT, 75 mg, qd	3 months/NR	TER, MMP-3, IL-6, and SSTCM
	Zheng Tao, 2019	TCM-5,250 ml,bid	CC_1_, 100 mg, bid	1 month/NR	TER, VAS, and WOMAC
	Yuan Fang, 2018	TCM-6,200 ml, bid	GSC, 0.5 g, tid	3 months/3 months	TER, VAS, WOMAC, SSTCM, ESR, and CRP
	Li Zhimin, 2018	TCM-7,250 ml, bid + *C*	DRC + CC_1_, 1 tablet, bid	8 weeks/NR	TER, Lequesne, VAS, WOMAC, IL-1, TNF-*α*, IL-6, and MMP-3
	Li Linzhong, 2014	TCM-8, qd + *C*	CC_2_, 100 mg, bid	6 months/NR	TER, WOMAC, and IL-6
	Li Ping, 2019	TCM-9,150 ml, bid + routine treatment	CC_1_, 0.2 g, qd + routine treatment	4 weeks/NR	TER, WOMAC, TNF-*α*, IL-1, and SS
	Liu Sheng, 2019	TCM-10,150 ml, bid	GSC, 0.5 g, bid	4 weeks/6 months	TER, VAS, WOMAC, MMP-3, RR, and SS

EUTCM	Yu Ming, 2018	TCMI-1,30 min, qd + *C*	AD + routine treatment	2 weeks/NR	TER, VAS, WOMAC, IL-1, TNF-*α*, Lysholm, and TGF-*β*
	Li Meng, 2017	TCMI, 30 min, qd + *C*	AD	12 months/NR	VAS, IL-1, TNF-*α*, TGF-*β*, and Lysholm
	Lei Shoubin, 2014	TCMI-2,30 min, bid + *C*	SH, 20 mg, qw	5 weeks/NR	TER, WOMAC, IL-1, and TNF-*α*
	Zeng Jiaofei, 2014	TCMI-3,30 min, bid	SH, 2.5 ml, qw	4 weeks, 5 weeks/NR	TER, VAS, IL-1, TNF-*α*, and Lysholm
	He Junlei, 2016	TCMI-4,30 min, qd + *C*	MLFET + MT	8 weeks/NR	TER, VAS, and WOMAC
	Chen Xi, 2015	TCMI-5,20 min, qd + TCMT	GC, 0.628 g, tid, po	14 days/NR	TER, IL-1, MMP-3, Lequesne, and SOD
	Zhou Gang, 2018	FWP-1,20-30 min, bid + FPR, bid	BW, 20–30 min, bid, ext + DDO, 2g, bid	2 weeks/NR	TER, TNF-*α*, WOMAC, and SS
	Ding Liming, 2016	FWP-2,10 min, bid + AD	AD	2 weeks/NR	MMP-3, VAS, and Lysholm
	Zhang Yanzhen, 2018	FWP-3, 20 min, qd	DDO, 0.9 g, tid, ext	2 weeks/NR	TER, WOMAC, Lequesne, and SSTCM
	Zhang Zhi, 2018	FWP-4,30 min, bid + IHN	DW, 30 min, bid, ext + IHN	2 weeks/NR	TER, MMP-3, VAS, and Lysholm
	Chen Hongmei, 2017	FWP-5, biw + *C*	ESWT, biw	8 weeks/NR	TER, IL-6, TNF-*α*, VAS, and Lysholm
	Zhang Hui, 2016	FWP-6,30 min, bid	GL, 30 min, bid, ext	2 weeks/NR	TER, WOMAC, VAS, and SSTCM
	Liu Lin, 2018	FWP-7,30 min, qd + *C*	GS, 0.314 mg, tid,po	15 days/NR	TER, CRP, ESR, SOD, and SSTCM
	Cui Shuping, 2016	FWP-8,30-60 min, bid + SH, qw, ia + QZP, ext	LST, 60 mg, tid, po + SH,qw,ia	3 weeks/NR	TER, CRP, ESR, and VAS
	Wang Tao, 2017	FWP-9, qd + GHC, 0.75 g, bid, po	NSRC, 0.5 g,qd + GHC, 0.75 g, bid	2 weeks/NR	TER, TNF-*α*, IL-1, and TGF-*β*
	Liu Enxiong, 2016	EAP-1, 10 min, tid + *C*	GHC, 750 mg, bid, po	42 days/NR	TER, IL-1, TNF-*α*, VAS, and Lequesne
	Liu Gui, 2017	EAP-2,20 min, tid + *C*	GS, 0.628 g, tid, po	12 weeks/NR	TER, MMP-3, IL-1, and TNF-*α*
	Liu Yongyu, 2014	SO, qd, ext + *C*	SH, qw, ia	35 days/NR	SS, IL-1, IL-6, TNF-*α*, WOMAC, and Lysholm
	Guo Wenhui, 2018	EAP-3,30 min, qd + *C*	OI, 15 ml, qw	4 weeks/NR	TER, CRP, ESR, IL-1, TNF-*α*
	Wang Yuan, 2018	EAP-4,8h,bid + XC, 1.5 mg, tid, po	GS, 0.628 mg,tid,po	2 weeks/NR	TER, SOD, WOMAC, VAS, and Lequesne

ACU	Chen Juan, 2017	WAC, qd + *C*	SH, injection, qw	4 weeks/NR	TER, VAS, Lysholm, and TNF-*α*
	Deng Jingming, 2015	WAC, tiw	ISRC, po, bid	4 weeks/NR	TER, VAS, and Lysholm
	Ding Minghui, 2009	WAC, qd	B	2 weeks/NR	TER and WOMAC
	Guo Qian, 2019	WAC, qd	B	3 weeks/NR	WOMAC, IL-1, TNF-*α*, IGF-1, FGF-2, and TGF-*β*
	Li Jianwu, 2008	WAC, qd	XGC, po, bid	4 weeks/NR	TER and VAS
	Li Jiangming, 2016	WAC + PCB	Routine Treatment	3 months/NR	WOMAC
	Liang Chao, 2016	WAC, qd	GHT, po, tid	4 weeks/NR	TER
	Lin Ruyi, 2019	WAC, qd	DSC, po, bid	4 weeks/NR	WOMAC, OPG, MMP-3, FGF-2, VEGF, and Ang I
	Liu Jin, 2014	ACU + DSTC, tid, po	FSRT, po, qd	2 weeks/NR	TER, WOMAC, IL-1, and TNF-*α*
	Luo Falan, 2018	ACU, qd + *C*	CC_1_, po, qd	8 weeks/NR	TER, IGF-1, RANKL, BGP, and OPG
	Wang Lina, 2018	WAC, qd + *C*	CC_1_, po, qd	4 weeks/NR	TER, WOMAC, IL-1, TNF-*α*, and IL-6
	Wang Xiaoling, 2017	WAC, qd	CC_1_, 200 mg,po	3 weeks/NR	WOMAC
	Xu Chen, 2018	WAC + APP, qd	SH, qw or GHT, po, bid	4 weeks/3 months	TER, CRP, ESR, and RR
	Xu Yahong, 2016	WAC + *C*, qd	USW, qd	1 month/NR	TER, VAS, WOMAC, Lysholm, IL-1, TNF-*α*, BGP, and OPG

AD, arthroscopic debridement; ACU, acupuncture; APP, auricular point pressing; Ang I, angiopoietin I; BW, boiled water; B, blank; bid, twice a day; BGP, Bone gla protein; BMP-7, bone morphogenetic protein-7; C, treatment of the control group; CC_1_, velecoxib capsule; CC_2_, Celebrex capsule; CRP, C-reactive protein; DW, distilled water; DDO, diclofenac diethylamine ointment; DRC, Divinegar Ruiyin capsule; DSESRC, diclofenac sodium enteric sustained release capsule; DSC, diclofenac sodium capsule; DSSRT, diclofenac sodium sustained release tablets; DSTC, dragon and soft-shelled turtle capsule; ext, external use; EUTCM, external use of traditional Chinese medicines; ESWT, extracorporeal shock wave therapy; EAP, external application prescription; ESR, erythrocyte sedimentation rate; FSRT, futalin sustained release tablets; FWP, fumigation and washing prescription; FGF-2, fibroblast growth factor-2; GC, glucosamine capsule; GL, Guyouling liniment; GS, glucosamine sulfate; GSC, glucosamine sulfate capsule; GHC, glucosamine hydrochloride capsule; GHT, glucosamine hydrochloride tablets; IL, interleukin; IGF-1, insulin-like growth factor 1; IHN, internal heat needle; ISRC, ibuprofen sustained release capsule; ia, intra-articular injection; LST, Loxoprofen sodium tablets; MLFET, middle- and low-frequency electric therapy apparatus; MT, mobilization technique; MMP, matrix metalloproteinase; NSRC, naproxen sustained release capsule; NR, no report.; OATCM, oral administration of traditional Chinese medicines; OI, ozone injection; OPG, osteoprotegerin; po, oral administration; QZP, Qizhu Zhanjin Powders; qd, once a day; qw, once a week; RANKL, receptor activator of nuclear factor-*κ* B ligand; RR: recurrence rate; SH, sodium hyaluronate; SO, Shangbai ointment; SSTCM, syndrome score of traditional Chinese medicine; SOD, superoxide dismutase; SH, sodium hyaluronate; SS, swelling score; tid, three times a day; TCM, traditional Chinese medicine; TER, total effective rate; TNF-*α*, tumor necrosis factor-*α*; TGF-*β*, transforming growth factor-*β*; TCMI, traditional Chinese medicine iontophoresis; traditional Chinese medicine treatment; USW, ultrashort wave; VAS, visual analogue scale; VEGF, vascular endothelial growth factor; WAC, warm acupuncture; WOMAC, Western Ontario and McMaster University osteoarthritis index; XC, Xinfeng capsule; XGC, Xianling Gubao capsule.

## Data Availability

The data used to support the findings of this study are included within the supplementary information files and the article

## Supplementary Materials

Supplementary Materials ESR and CRP are indicators of inflammatory activity in the body; Figure S1 contains the forest plot of ESR and CRP with TCM therapy and Western medicine therapy; Figure S1-A is the plot of ESR, and Figure S1–B is the plot of CRP. Table S1: the prescriptions of TCMs involved in the OATCM and EUTCM; Table S2: acupoints involved in the treatment of OA by ACU; Table S3: international coding corresponding to acupoints; Table S4 : TCM therapy vs. Western medicine therapy on self-activity score; Table S5 : TCM therapy vs. Western medicine therapy on inflammatory cytokines; Table S6: the level of bone metabolism indexes of TCM therapy vs. Western medicine therapy; Table S7 : ACU treatment of TCM therapy vs. Western medicine therapy on vascular function factors; and Table S8: TCM therapy vs. Western medicine therapy on RR and SOD.
