# Supplementary material for: Evaluation of the Therapeutic Effect of Traditional Chinese Medicine on Osteoarthritis: A Systematic Review and Meta-Analysis
Source: Pain Res Manag. 2020 Dec 14;2020:5712187. doi: 10.1155/2020/5712187 (PMC7752303; doi:10.1155/2020/5712187)
Supplement: Supplementary Materials — ESR and CRP are indicators of inflammatory activity in the body; Figure S1 contains the forest plot of ESR and CRP with TCM therapy and Western medicine therapy; Figure S1-A is the plot of ESR, and Figure S1–B is the plot of CRP. Table S1: the prescriptions of TCMs involved in the OATCM and EUTCM; Table S2: acupoints involved in the treatment of OA by ACU; Table S3: international coding corresponding to acupoints; Table S4 : TCM therapy vs. Western medicine therapy on self-activity score; Table S5 : TCM therapy vs. Western medicine therapy on inflammatory cytokines; Table S6: the level of bone metabolism indexes of TCM therapy vs. Western medicine therapy; Table S7 : ACU treatment of TCM therapy vs. Western medicine therapy on vascular function factors; and Table S8: TCM therapy vs. Western medicine therapy on RR and SOD. [file 5712187.f1.zip › 5712187.f1/Table S1.docx]

**Table S1.** The prescriptions of TCMs involved in the OATCM and EUTCM.

| **Study ID** | **Drugs** | **Composition of TCMs** | **An added or subtracted prescription** | **Ancient prescription** |
| --- | --- | --- | --- | --- |
| Zhou Gang 2018 | FWP-1 | Angeticae Sinensis Radix, Dipsacus asperoides C. Y. Cheng et T. M. Ai, Eucommiae Cortex, Chuanxiong Rhizoma,Cibotii Rhizoma, Drynariae Rhizoma, Cyathulae Radix, Carthami Flos, Persicae Semen, Paeoniae Radix Alba, Eupolyphaga Steleophaga, Lycii Cortex, Pyritum, Astragali Radix | Xuefu Zhuyu Decoction | *Correction on Errors in Medical Classics* |
| Ding Liming 2016 | FWP-2 | Carthami Flos , Arnebiae Radix, Angeticae Sinensis Radix, Angelieae Pubescentis Radix, Saposhnikoviae Radix, Lycopodii Herba, Piperis Albae Fructus, Siphonostegiae Herba, Caulis Impatientis, Sappan Lignum | Duhuo Jisheng Decoction | *Important Prescriptions Worth a Thousand Gold for Emergency* |
| Zhang Yanzhen 2018 | FWP-3 | Angelieae Pubescentis Radix, Dipsacus asperoides C. Y. Cheng et T. M. Ai, Achyranthis Bidentatae Radix, Clematis chinensis Osbeck, Smilacis Glabrae Rhizoma, Paeoniae Radix Rubra, Gentianae macrophyllae Radix, Saposhnikoviae Radix, Sinomenii Caulis, Zanthoxyli Pericarpium, Artemisiae Argyi Folium, Chuanxiong Rhizoma | Qin Jiao Powder | *Golden Mirror of Medicine* |
| Zhang Zhi 2018 | FWP-4 | Rhei Radix et Rhizoma, Angelicae Dahuricae Radix, Curcumae Longae Rhizoma, Atractylodis Rhizoma, Zanthoxyli Pericarpium, Arisaematis Rhizoma, Phellodendri Chinensis Cortex, Borneolum Syntheticum, Aconiti Radix, Aconiti Kusnezoffii Radix , Caryophyllus Flos, Tripterygium wilfordii Hook. f | Dahuoluo Pill | *Pharmacopoeia of the people's Republic of China* |
| Chen Hongmei 2017 | FWP-5 | Spatholobi Caulis, Erythrina variegata L., Chuanxiong Rhizoma, Lycopodii Herba, Clematis chinensis Osbeck, Aristolochia mollissima Hance, Mori Ramulus Tostum, Cinnamomi Ramulus, Carthami Flos, Artemisiae Argyi Folium, Myrrha Tostum, Liquidambaris Fructus, Angeticae Sinensis Radix, Corydalis Rhizoma, Olibanum | Haitongpi Decoction | *Golden Mirror of Medicine* |
| Zhang Hui 2016 | FWP-6 | Liquidambaris Fructus, Lycopodii Herba, Ephedrae Herba, Aconiti Radix Cocta, Achyranthis Bidentatae Radix, Caulis Impatientis, Clematis chinensis Osbeck, Schizonepetae Herba, Myrrha, Olibanum, Cinnamomi Ramulus, Zanthoxyli Pericarpium, Saposhnikoviae Radix, Aconiti Lateralis Radix Praeparata, Stephaniae Tetrandrae Radix, Gentianae macrophyllae Radix | Unknown | Unknown |
| Liu Lin 2018 | FWP-7 | Zanthoxyli Pericarpium, Cinnamomi Ramulus, Caulis Impatientis, Angelicae Dahuricae Radix, Foeniculi Fructus, Carthami Flos, Acanthopanacis Cortex, Acori Tatarinowii Rhizoma | Wujiapi decoction | *Treatise on Three Categories of Pathogenic Factors* |
| Cui Shuping 2016 | FWP-8 | Erythrina variegata L., Caulis Impatientis, Olibanum, Myrrha, Angeticae Sinensis Radix, Zanthoxyli Pericarpium, Saposhnikoviae Radix, Carthami Flos, Chuanxiong Rhizoma, Clematis chinensis Osbeck, Angelicae Dahuricae Radix, Glycyrrhiza Radix et Rhizoma | Haitongpi Decoction | *Golden Mirror of Medicine* |
| Wang Tao 2017 | FWP-9 | Aconiti Radix Cocta, Angelieae Pubescentis Radix, Achyranthis Bidentatae Radix, Chuanxiong Rhizoma, Eucommiae Cortex, Lycopodii Herba, Chaenomelis Fructus, Eupolyphaga Steleophaga, Zaocys, Clematis chinensis Osbeck, Glycyrrhiza Radix et Rhizoma | Duhuo Jisheng Decoction | *Important Prescriptions Worth a Thousand Gold for Emergency* |
| Liu Enxiong 2016 | EAP-1 | Lycopodii Herba , Corydalis Rhizoma, Cyathulae Radix, Caulis Impatientis, Chaenomelis Fructus, Salviae Mihiorrhizae Radix et Rhizoma | Shenjincao Lotion | *Zhao Bingnan Clinical experience Collection* |
| Liu Gui 2017 | EAP-2 | Rehmanniae Radix Praeparata, Eucommiae Cortex, Angelieae Pubescentis Radix, Taxilli Herba, Drynariae Rhizoma, Epimedii Herba, Achyranthis Bidentatae Radix, Clematis chinensis Osbeck, Typhonii Rhizoma, Sinapis Albae Semen, Spatholobi Caulis, Glycyrrhiza Radix et Rhizoma | Duhuo Jisheng Decoction | *Important Prescriptions Worth a Thousand Gold for Emergency* |
| Liu Yongyu 2014 | SO | Bletillae Rhizoma, Carthami Flos, Rhei Radix et Rhizoma, Platycladi Cacumen, Lycopi Herba | Shuangbai powder ointment | *Traditional Chinese Traumatology* |
| Guo Wenhui 2018 | EAP-3 | Cyathulae Radix, Aconiti Radix , Chuanxiong Rhizoma, Rhei Radix et Rhizoma, Dipsaci Radix, Taxilli Herba, Carthami Flos, Notoginseng Radix, Clematis chinensis Osbeck, Angelieae Pubescentis Radix | Duhuo Jisheng Decoction | *Important Prescriptions Worth a Thousand Gold for Emergency* |
| Wang Yuan 2018 | EAP-4 | Salviae Mihiorrhizae Radix et Rhizoma, Olibanum, Myrrha, Chuanxiong Rhizoma, Piperis Longi Fructus, Notoginseng Radix et Rhizoma | Ruxiang Moyao power | *Preions for Universal Relief* |
| Wang Zhenhua 2018 | TCM-1 | Eucommiae Cortex, Taxilli Herba, Dipsacus asperoides C. Y. Cheng et T. M. Ai, Angelieae Pubescentis Radix, Morindae Officinalis Radix, Epimedii Herba, Achyranthis Bidentatae Radix, Salviae Mihiorrhizae Radix et Rhizoma, Rehmanniae Radix Praeparata, Drynariae Rhizoma, Bungarus Parvus, Aconiti Radix Cocta | Duhuo Jisheng Decoction | *Important Prescriptions Worth a Thousand Gold for Emergency* |
| Kuang Yao 2018 | TCM-2 | Angelieae Pubescentis Radix, Taxilli Herba, Angeticae Sinensis Radix, Poria, Codonopsis Radix, Rehmanniae Radix Praeparata, Saposhnikoviae Radix, Gentianae macrophyllae Radix, Paeoniae Radix Alba,Cyathulae Radix, Chuanxiong Rhizoma,Aconiti Radix Cocta, Asari Radix et Rhizoma, Glycyrrhiza Radix et Rhizoma | Duhuo Jisheng Decoction | *Important Prescriptions Worth a Thousand Gold for Emergency* |
| Wen Yangyang 2019 | TCM-3 | Rehmanniae Radix, Psoraleae Fructus, Astragali Radix ,Angeticae Sinensis Radix, Spatholobi Caulis, Chuanxiong Rhizoma, Cistanches Herba, Dipsaci Radix, Liquidambaris Fructus, Citri Reticulatae Pericarpium, Salviae Mihiorrhizae Radix et Rhizoma, Cibotii Rhizoma | Unknown | Unknown |
| Cui Hongfang 2018 | TCM-4 | Angeticae Sinensis Radix, Rehmanniae Radix Praeparata, Pheretima, Achyranthis Bidentatae Radix, Eucommiae Cortex, Clematis chinensis Osbeck, Dipsaci Radix, Cuscutae Semen, Sambucus chinensis, Codonopsis Radix, Glycyrrhiza Radix et Rhizoma Praeparata cum Melle | Unknown | Unknown |
| Zheng Tao 2019 | TCM-5 | Rehmanniae Radix Praeparata, Eucommiae Cortex, Dipsaci Radix, Psoraleae Fructus, Olibanum, Myrrha, Draconis Sanguis, Angeticae Sinensis Radix, Taxilli Herba | Unknown | Unknown |
| Yuan Fang 2018 | TCM-6 | Dipsaci Radix, Drynariae Rhizoma, Achyranthis Bidentatae Radix, Spatholobi Caulis, Homalomenae Rhizoma, Taxilli Herba, Pini Lignum Nodi, Eupolyphaga Steleophaga | Huoluo Xiaolingdan pills | *Reference to the West in Medicine* |
| Li Zhimin 2018 | TCM-7 | Achyranthis Bidentatae Radix, Dipsaci Radix, Eucommiae Cortex, Epimedii Herba, Cibotii Rhizoma, Astragali Radix, Rehmanniae Radix Praeparata, Clematis chinensis Osbeck, Notopterygii Rhizoma et Radix, Angelieae Pubescentis Radix, Gentianae macrophyllae Radix, Angeticae Sinensis Radix, Chuanxiong Rhizoma, Myrrha, Persicae Semen, Carthami Flos, Pheretima, Hirudo, Codonopsis Radix, Glycyrrhiza Radix et Rhizoma Praeparata cum Melle | Shentong Zhuyu Decoction | *Correction on Errors in Medical Classics* |
| Li Linzhong 2014 | TCM-8 | Eucommiae Cortex, Rehmanniae Radix Praeparata, Corydalis Rhizoma, Spatholobi Caulis, Achyranthis Bidentatae Radix, Sparganii Rhizoma, Curcumae Rhizoma, Curculiginis Rhizoma, Salviae Mihiorrhizae Radix et Rhizoma, Aconiti Lateralis Radix Praeparata, Buthotoxin, Morindae Officinalis Radix, Carthami Flos | Guishen pill | *Jingyue’s Complete Works* |
| Li Ping 2019 | TCM-9 | Poria, Paeoniae Radix Alba, Zingiberis Rhizoma Recens, Atractylodis Macrocephalae Rhizoma, Notopterygii Rhizoma et Radix, Gentianae macrophyllae Radix, Angeticae Sinensis Radix, Achyranthis Bidentatae Radix, Taxilli Herba, Astragali Radix, Olibanum, Glycyrrhiza Radix et Rhizoma | ZhenWu Decoction | *Treatise on Cold Pathogenic Diseases* |
| Liu Sheng 2019 | TCM-10 | Angelieae Pubescentis Radix, Dipsaci Radix, Eucommiae Cortex, Ramulus Cinnamomi, Saposhnikoviae Radix, Chuanxiong Rhizoma, Cyathulae Radix, Asari Radix et Rhizoma, Gentianae macrophyllae Radix, Poria, Ginseng Radix, Angeticae Sinensis Radix, Paeoniae Radix Alba, Rehmanniae Radix Praeparata, Lycii Cortex, Glycyrrhiza Radix et Rhizoma | Duhuo Xuduan Decoction | *The secret of the outside station* |
| Yu Ming 2018 | TCMI-1 | Artemisiae Argyi Folium, Angeticae Sinensis Radix, Taxilli Herba, Achyranthis Bidentatae Radix, Siphonostegiae Herba, Angelieae Pubescentis Radix, Gentianae macrophyllae Radix, Drynariae Rhizoma, Liquidambaris Fructus, Acanthopanacis Cortex, Polygoni Cuspidati Rhizoma, Zanthoxyli Pericarpium, Typhonii Rhizoma, Zingiberis Rhizoma, Carthami Flos, Lycopodii Herba, Asari Radix et Rhizoma | Unknown | *Unknown* |
| Lei Shoubin 2014 | TCMI-2 | Carthami Flos, Clematis chinensis Osbeck, Lycopodii Herba, Taraxaci Herba, Artemisiae Argyi Folium, Saposhnikoviae Radix, Schizonepetae Herba, Myrrha, Eucommiae Cortex | Tongluo Zhitong Powder | *Unknown* |
| Zeng Jiaofei 2014 | TCMI-3 | Lonicerae Flos, Angelicae Dahuricae Radix, Saposhnikoviae Radix, Bulbus Fritillariae Thunbergii, Trichosanthis Radix, Angeticae Sinensis Radix, Olibanum, Paeoniae Radix Rubra, Myrrha, Citri Reticulatae Pericarpium, pangolin scales, Gleditsiae Spina, Glycyrrhiza Radix et Rhizoma, Aconiti Radix Cocta, Strychni Semen | Xianfang Huoming Infection | *Complete Effective Preions for Women’s Diseases* |
| He Junlei 2016 | TCMI-4 | Astragali Radix, Saposhnikoviae Radix, Angeticae Sinensis Radix, Notopterygii Rhizoma et Radix, Aconiti Lateralis Preparata Radix, Glycyrrhiza Radix et Rhizoma, Curcumae Longae Rhizoma | Juanbi Decoction | *The prescription of Yang's Family's Private Collection* |
| Chen Xi 2015 | TCMI-5 | Astragali Radix, Poria, Atractylodis Macrocephalae Rhizoma, Coicis Semen, Citri Reticulatae Pericarpium, Pinelliae Preparata Rhizome, Eucommiae Cortex, Drynariae Rhizoma, Angelieae Pubescentis Radix, Chaenomelis Fructus, Dipsacus asperoides C. Y. Cheng et T. M. Ai, Cyathulae Radix, Achyranthis Bidentatae Radix, Salviae Mihiorrhizae Radix et Rhizoma, Persicae Semen, Carthami Flos, Lycopi Herba, Spatholobi Caulis, Glycyrrhiza Radix et Rhizoma | Shenjin Decoction | *Zhao Bingnan Clinical Experience Collection* |
