# Supplementary material for: Evaluation of the Therapeutic Effect of Traditional Chinese Medicine on Osteoarthritis: A Systematic Review and Meta-Analysis
Source: Pain Res Manag. 2020 Dec 14;2020:5712187. doi: 10.1155/2020/5712187 (PMC7752303; doi:10.1155/2020/5712187)
Supplement: Supplementary Materials — ESR and CRP are indicators of inflammatory activity in the body; Figure S1 contains the forest plot of ESR and CRP with TCM therapy and Western medicine therapy; Figure S1-A is the plot of ESR, and Figure S1–B is the plot of CRP. Table S1: the prescriptions of TCMs involved in the OATCM and EUTCM; Table S2: acupoints involved in the treatment of OA by ACU; Table S3: international coding corresponding to acupoints; Table S4 : TCM therapy vs. Western medicine therapy on self-activity score; Table S5 : TCM therapy vs. Western medicine therapy on inflammatory cytokines; Table S6: the level of bone metabolism indexes of TCM therapy vs. Western medicine therapy; Table S7 : ACU treatment of TCM therapy vs. Western medicine therapy on vascular function factors; and Table S8: TCM therapy vs. Western medicine therapy on RR and SOD. [file 5712187.f1.zip › 5712187.f1/Table S2.docx]

**Table S2.** Acupoints involved in the treatment of OA by ACU.

| **Study ID** | **Acupoint** |
| --- | --- |
| Luo Falan 2018 | Liangqiu, Xiyan, Xuehai, Heding, Yanglingquan, Yinlingquan |
| Xu Chen 2018 | Xiyan, Dubi, Xuehai, Yanglingquan, Yinlingquan |
| Xu Yahong 2016 | Xiyan, Heding, Weizhong, Xuehai, Zusanli, Yanglingquan, Ashi |
| Ding Minghui 2009 | Xuehai, Xiyan, Zusanli, Sanyinjiao, Shenyu, Dachangyu, Piyu |
| Wang Lina 2018 | Liangqiu, Xuehai, Xiyan, Yanglingquan, Yinlingquan, Zusanli, Heding, Sanyinjiao |
| Chen Juan 2017 | Xiyan, Liangqiu, Heding, Xuehai, Yanglingquan, Ashi, Zusanli, Guanyuan, Qihai |
| Wang XiaoLing 2017 | Dubi, Xiyan, Xuehai, Liangqiu, Yanglingquan, Yinlingquan, Fengshi, Weizhong, Heyang |
| Lin Ruyi 2019 | Xiyan, Yanglingquan, Yinlingquan, Liangqiu, Zusanli |
| Guo Qian 2019 | Kuangu, Xiyan, Heding |
| Deng Jingming 2015 | Xiyan, Dubi, Ququan, Heding, Xiyangguan, Ashi |
| Liu Jin 2014 | Heding, Liangqiu, Xuehai, Dubi, Yanglingquan, Zusanli, Fengshi, Xiyangguan, Ashi |
| Li Jianwu 2018 | Dubi, Xiyan, Zusanli |
| Liang Chao 2016 | Xuehai, Liangqiu, Xiyan, Yanglingquan, Yinlingquan, Ququan |
