# Supplementary material for: Evaluation of the Therapeutic Effect of Traditional Chinese Medicine on Osteoarthritis: A Systematic Review and Meta-Analysis
Source: Pain Res Manag. 2020 Dec 14;2020:5712187. doi: 10.1155/2020/5712187 (PMC7752303; doi:10.1155/2020/5712187)
Supplement: Supplementary Materials — ESR and CRP are indicators of inflammatory activity in the body; Figure S1 contains the forest plot of ESR and CRP with TCM therapy and Western medicine therapy; Figure S1-A is the plot of ESR, and Figure S1–B is the plot of CRP. Table S1: the prescriptions of TCMs involved in the OATCM and EUTCM; Table S2: acupoints involved in the treatment of OA by ACU; Table S3: international coding corresponding to acupoints; Table S4 : TCM therapy vs. Western medicine therapy on self-activity score; Table S5 : TCM therapy vs. Western medicine therapy on inflammatory cytokines; Table S6: the level of bone metabolism indexes of TCM therapy vs. Western medicine therapy; Table S7 : ACU treatment of TCM therapy vs. Western medicine therapy on vascular function factors; and Table S8: TCM therapy vs. Western medicine therapy on RR and SOD. [file 5712187.f1.zip › 5712187.f1/Table S3.docx]

**Table S3.** International coding corresponding to acupoints.

| **Acupoint** | **International coding** | **Acupoint** | **International coding** |
| --- | --- | --- | --- |
| Xiyan | EX-LE4 | Ququan | LR8 |
| Yanglingquan | GB34 | Xiyangguan | GB33 |
| Zusanli | ST36 | Shenyu | BL23 |
| Xuehai | SP10 | Dachangyu | BL25 |
| Heding | EX-LE2 | Piyu | BL20 |
| Dubi | ST35 | Guanyuan | CV4 |
| Liangqiu | ST34 | Qihai | CV6 |
| Yinlingquan | ST9 | Heyang | BL55 |
| Ashi |  | Kuangu | EX-LE1 |
| Weizhong | BL40 |  |  |
| Sanyinjiao | SP9 |  |  |
| Fengshi | GB31 |  |  |
