# Supplementary material for: Evaluation of the Therapeutic Effect of Traditional Chinese Medicine on Osteoarthritis: A Systematic Review and Meta-Analysis
Source: Pain Res Manag. 2020 Dec 14;2020:5712187. doi: 10.1155/2020/5712187 (PMC7752303; doi:10.1155/2020/5712187)
Supplement: Supplementary Materials — ESR and CRP are indicators of inflammatory activity in the body; Figure S1 contains the forest plot of ESR and CRP with TCM therapy and Western medicine therapy; Figure S1-A is the plot of ESR, and Figure S1–B is the plot of CRP. Table S1: the prescriptions of TCMs involved in the OATCM and EUTCM; Table S2: acupoints involved in the treatment of OA by ACU; Table S3: international coding corresponding to acupoints; Table S4 : TCM therapy vs. Western medicine therapy on self-activity score; Table S5 : TCM therapy vs. Western medicine therapy on inflammatory cytokines; Table S6: the level of bone metabolism indexes of TCM therapy vs. Western medicine therapy; Table S7 : ACU treatment of TCM therapy vs. Western medicine therapy on vascular function factors; and Table S8: TCM therapy vs. Western medicine therapy on RR and SOD. [file 5712187.f1.zip › 5712187.f1/Table S4.docx]

**Table S4.** TCM Therapy *vs.* Western Medicine Therapy on self-activity score.

| **self-activity score** | **Treatment mode** | **Number of**  **studies** | **Study ID** | **Cases of**  **experimental group** | **Cases of**  **control group** | **MD [95%CI]** | **Z-value** | ***P*-value** | **Effect model** |
| --- | --- | --- | --- | --- | --- | --- | --- | --- | --- |
| SS | OATCM | 3 | Kuang Yao 2018  Li Ping 2019  Liu Sheng 2019 | 162 | 152 | -0.26 [-0.40, -0.13] | 3.90 | < 0.0001 | Random |
|  | EUTCM | 2 | Zhou Gang 2018  Liu Yongyu 2014 | 68 | 68 | -0.77 [-2.14, 0.60] | 1.11 | 0.27 |  |
| Lequesne | OATCM | 1 | Li Zhimin 2018 | 48 | 48 | -2.43 [-3.27, -1.59] | 5.66 | < 0.00001 | Random |
|  | EUTCM | 4 | Chen Xi 2015  Zhang Yanzhen 2018  Liu Enxiong 2016  Wang Yuan 2018 | 172 | 172 | -0.90 [-1.34, -0.47] | 4.04 | <0.0001 |  |
