# Supplementary material for: Evaluation of the Therapeutic Effect of Traditional Chinese Medicine on Osteoarthritis: A Systematic Review and Meta-Analysis
Source: Pain Res Manag. 2020 Dec 14;2020:5712187. doi: 10.1155/2020/5712187 (PMC7752303; doi:10.1155/2020/5712187)
Supplement: Supplementary Materials — ESR and CRP are indicators of inflammatory activity in the body; Figure S1 contains the forest plot of ESR and CRP with TCM therapy and Western medicine therapy; Figure S1-A is the plot of ESR, and Figure S1–B is the plot of CRP. Table S1: the prescriptions of TCMs involved in the OATCM and EUTCM; Table S2: acupoints involved in the treatment of OA by ACU; Table S3: international coding corresponding to acupoints; Table S4 : TCM therapy vs. Western medicine therapy on self-activity score; Table S5 : TCM therapy vs. Western medicine therapy on inflammatory cytokines; Table S6: the level of bone metabolism indexes of TCM therapy vs. Western medicine therapy; Table S7 : ACU treatment of TCM therapy vs. Western medicine therapy on vascular function factors; and Table S8: TCM therapy vs. Western medicine therapy on RR and SOD. [file 5712187.f1.zip › 5712187.f1/Table S5.docx]

**Table S5.** TCM Therapy *vs.* Western Medicine Therapy on Inflammatory cytokines.

| **self-activity score** | **Treatment mode** | **Number of**  **studies** | **Study ID** | **Cases of**  **experimental group** | **Cases of**  **control group** | **MD [95%CI]** | **Z-value** | ***P*-value** | **Effect model** |
| --- | --- | --- | --- | --- | --- | --- | --- | --- | --- |
| IL-6 | OATCM | 4 | Li Zhimin 2018  Cui Hongfang 2018  Li Linzhong 2014  Wang Zhenhua 2018 | 202 | 202 | -16.44 [-37.43, 4.55] | 1.53 | 0.12 | Random |
|  | EUTCM | 2 | Chen Hongmei 2017 Liu Yongyu 2017 | 61 | 61 | -2.64 [-4.63, -0.65] | 2.61 | 0.009 |  |
|  | ACU | 1 | Wang Lina 2018 | 59 | 59 | -8.56 [-11.60, -5.52] | 5.51 | < 0.00001 |  |
| TGF-β | OATCM | 1 | Wen Yangyang 2019 | 63 | 63 | 133.52 [112.03, 155.01] | 12.18 | < 0.00001 | Random |
|  | EUTCM | 3 | Li Meng 2017  Wang Tao 2017  Yu Ming 2018 | 133 | 133 | 12.33 [0.96, 23.70] | 2.13 | 0.03 |  |
|  | ACU | 1 | Guo Qian 2019 | 79 | 79 | 9.21 [7.85, 10.57] | 13.27 | < 0.00001 |  |
