# Supplementary material for: Evaluation of the Therapeutic Effect of Traditional Chinese Medicine on Osteoarthritis: A Systematic Review and Meta-Analysis
Source: Pain Res Manag. 2020 Dec 14;2020:5712187. doi: 10.1155/2020/5712187 (PMC7752303; doi:10.1155/2020/5712187)
Supplement: Supplementary Materials — ESR and CRP are indicators of inflammatory activity in the body; Figure S1 contains the forest plot of ESR and CRP with TCM therapy and Western medicine therapy; Figure S1-A is the plot of ESR, and Figure S1–B is the plot of CRP. Table S1: the prescriptions of TCMs involved in the OATCM and EUTCM; Table S2: acupoints involved in the treatment of OA by ACU; Table S3: international coding corresponding to acupoints; Table S4 : TCM therapy vs. Western medicine therapy on self-activity score; Table S5 : TCM therapy vs. Western medicine therapy on inflammatory cytokines; Table S6: the level of bone metabolism indexes of TCM therapy vs. Western medicine therapy; Table S7 : ACU treatment of TCM therapy vs. Western medicine therapy on vascular function factors; and Table S8: TCM therapy vs. Western medicine therapy on RR and SOD. [file 5712187.f1.zip › 5712187.f1/Table S6.docx]

**Table S6.** The level of Bone Metabolism Indexes of TCM Therapy *vs.* Western Medicine Therapy.

| **self-activity score** | **Treatment mode** | **Number of**  **studies** | **Study ID** | **Cases of**  **experimental group** | **Cases of**  **control group** | **MD [95%CI]** | **Z-value** | ***P*-value** | **Effect model** |
| --- | --- | --- | --- | --- | --- | --- | --- | --- | --- |
| IGF-1 | ACU | 2 | Guo Qian 2019  Luo Falan 2018 | 121 | 121 | 8.58 [-5.83, 22.98] | 1.17 | 0.24 | Random |
| OPG | ACU | 3 | Lin Ruyi 2019  Luo Falan 2018  Xu Yahong 2016 | 139 | 139 | 0.28 [-0.85, 1.42] | 0.49 | 0.62 | Random |
| BGP | ACU | 3 | Luo Falan 2018  Xu Yahong 2016  Lin Ruyi 2019 | 139 | 139 | 1.12 [0.30, 1.93] | 2.69 | 0.007 | Random |
| FGF-2 | ACU | 2 | Guo Qian 2019  Lin Ruyi 2019 | 122 | 122 | 8.30 [5.92, 10.69] | 6.82 | <0.00001 | Random |
| RANKL | ACU | 1 | Luo Falan 2018 | 42 | 42 | -4.85 [-6.20, -3.50] | 7.03 | <0.00001 | / |
| BMP-7 | OATCM | 1 | Wang Zhenhua 2018 | 53 | 53 | -2.33 [-3.15, -1.51] | 5.57 | < 0.00001 | / |
