# Supplementary material for: Evaluation of the Therapeutic Effect of Traditional Chinese Medicine on Osteoarthritis: A Systematic Review and Meta-Analysis
Source: Pain Res Manag. 2020 Dec 14;2020:5712187. doi: 10.1155/2020/5712187 (PMC7752303; doi:10.1155/2020/5712187)
Supplement: Supplementary Materials — ESR and CRP are indicators of inflammatory activity in the body; Figure S1 contains the forest plot of ESR and CRP with TCM therapy and Western medicine therapy; Figure S1-A is the plot of ESR, and Figure S1–B is the plot of CRP. Table S1: the prescriptions of TCMs involved in the OATCM and EUTCM; Table S2: acupoints involved in the treatment of OA by ACU; Table S3: international coding corresponding to acupoints; Table S4 : TCM therapy vs. Western medicine therapy on self-activity score; Table S5 : TCM therapy vs. Western medicine therapy on inflammatory cytokines; Table S6: the level of bone metabolism indexes of TCM therapy vs. Western medicine therapy; Table S7 : ACU treatment of TCM therapy vs. Western medicine therapy on vascular function factors; and Table S8: TCM therapy vs. Western medicine therapy on RR and SOD. [file 5712187.f1.zip › 5712187.f1/Table S7.docx]

**Table S7.** ACU treatment of TCM Therapy *vs.* Western Medicine Therapy on Vascular Function Factors.

| **self-activity score** | **Number of**  **studies** | **Study ID** | **Cases of**  **experimental group** | **Cases of**  **control group** | **MD [95%CI]** | **Z-value** | **P-value** |
| --- | --- | --- | --- | --- | --- | --- | --- |
| Ang I | 1 | Lin Ruyi 2019 | 43 | 43 | -10.97 [-12.50, -9.44] | 14.05 | <0.00001 |
| VEGF | 1 | Lin Ruyi 2019 | 43 | 43 | -26.39 [-32.62, -20.16] | 8.31 | <0.00001 |
