# Supplementary material for: Evaluation of the Therapeutic Effect of Traditional Chinese Medicine on Osteoarthritis: A Systematic Review and Meta-Analysis
Source: Pain Res Manag. 2020 Dec 14;2020:5712187. doi: 10.1155/2020/5712187 (PMC7752303; doi:10.1155/2020/5712187)
Supplement: Supplementary Materials — ESR and CRP are indicators of inflammatory activity in the body; Figure S1 contains the forest plot of ESR and CRP with TCM therapy and Western medicine therapy; Figure S1-A is the plot of ESR, and Figure S1–B is the plot of CRP. Table S1: the prescriptions of TCMs involved in the OATCM and EUTCM; Table S2: acupoints involved in the treatment of OA by ACU; Table S3: international coding corresponding to acupoints; Table S4 : TCM therapy vs. Western medicine therapy on self-activity score; Table S5 : TCM therapy vs. Western medicine therapy on inflammatory cytokines; Table S6: the level of bone metabolism indexes of TCM therapy vs. Western medicine therapy; Table S7 : ACU treatment of TCM therapy vs. Western medicine therapy on vascular function factors; and Table S8: TCM therapy vs. Western medicine therapy on RR and SOD. [file 5712187.f1.zip › 5712187.f1/Table S8.docx]

**Table S8.** TCM Therapy *vs.* Western Medicine Therapy on RR and SOD.

| **self-activity score** | **Treatment mode** | **Number of**  **studies** | **Study ID** | **Cases of**  **experimental group** | **Cases of**  **control group** | **MD [95%CI]** | **Z-value** | ***P*-value** | **Effect model** |
| --- | --- | --- | --- | --- | --- | --- | --- | --- | --- |
| RR | ACU | 1 | Xu Chen 2018 | 40 | 20 | 0.03 [0.00, 0.22] | 3.31 | 0.0009 | fixed |
|  | OATCM | 3 | Wang Zhenhua 2018  Kuang Yao 2018  Liu Sheng 2019 | 134 | 124 | 0.35 [0.20, 0.64] | 3.47 | 0.0005 |  |
| SOD | OATCM | 1 | Wen Yangyang 2019 | 63 | 63 | 1.21 [0.93, 1.49] | 8.54 | < 0.00001 | Random |
|  | EUTCM | 3 | Chen Xi 2015  Liu Lin 2018  Wang Yuan 2018 | 110 | 110 | 20.93 [12.98, 28.88] | 5.16 | < 0.00001 |  |
